# Laparoscopic endoscopic cooperative surgery for gastric subepithelial lesion during laparoscopic sleeve gastrectomy for severe obesity

**DOI:** 10.1186/s40792-024-02027-0

**Published:** 2024-09-26

**Authors:** Takumi Miwa, Yuji Ishibashi, Fumihiko Hatao, Kohei Shimoji, Kazuhiro Imamura, Yasuhiro Morita

**Affiliations:** 1 Department of Surgery, Tokyo Metropolitan Tama Medical Center, 2-8-29 Musashidai, Fuchu-shi, Tokyo, 183-8524 Japan; 2 Department of Gastroenterology, Tokyo Metropolitan Tama Medical Center, Tokyo, Japan

**Keywords:** Sleeve gastrectomy, Laparoscopic endoscopic cooperative surgery, Subepithelial lesion

## Abstract

**Background:**

The frequency of pathologies detected incidentally before, during, and after a bariatric surgery, such as subepithelial lesion (SEL) of the stomach, is likely to rise as bariatric surgery becomes more common.

**Case presentation:**

A 49-year-old female patient presented with severe obesity, for which laparoscopic sleeve gastrectomy (LSG) was planned. During a preoperative examination, endoscopy revealed a 10 mm SEL in the posterior wall of the upper body of the stomach. Excision of the SEL was performed simultaneously with the LSG. Endoscopy demonstrated that the SEL was situated on the remnant side of the stomach. Endoscopic resection using laparoscopic endoscopic cooperative surgery was performed for the SEL, and the thinned gastric wall was closed by hand-sewing. Thereafter, LSG was performed. Pathological analysis of the SEL led to a diagnosis of leiomyoma. The patient was discharged on postoperative day 6.

**Conclusion:**

Surgeons should be prepared to manage incidentally detected pathologies before, during, and after bariatric surgery and to choose the surgical method most suitable to the individual patient.

## Background

The frequency of pathologies detected incidentally during laparoscopic bariatric surgery, such as subepithelial lesion (SEL), is currently estimated to be approximately 2% but is likely to rise as bariatric surgery becomes more common [1]. In such cases, the lesion may be excised during bariatric surgery. Herein, we report performing laparoscopic endoscopic cooperative surgery (LECS) for the excision of a gastric SEL during laparoscopic sleeve gastrectomy (LSG) for severe obesity.

## Case presentation

**Fig. 1:**
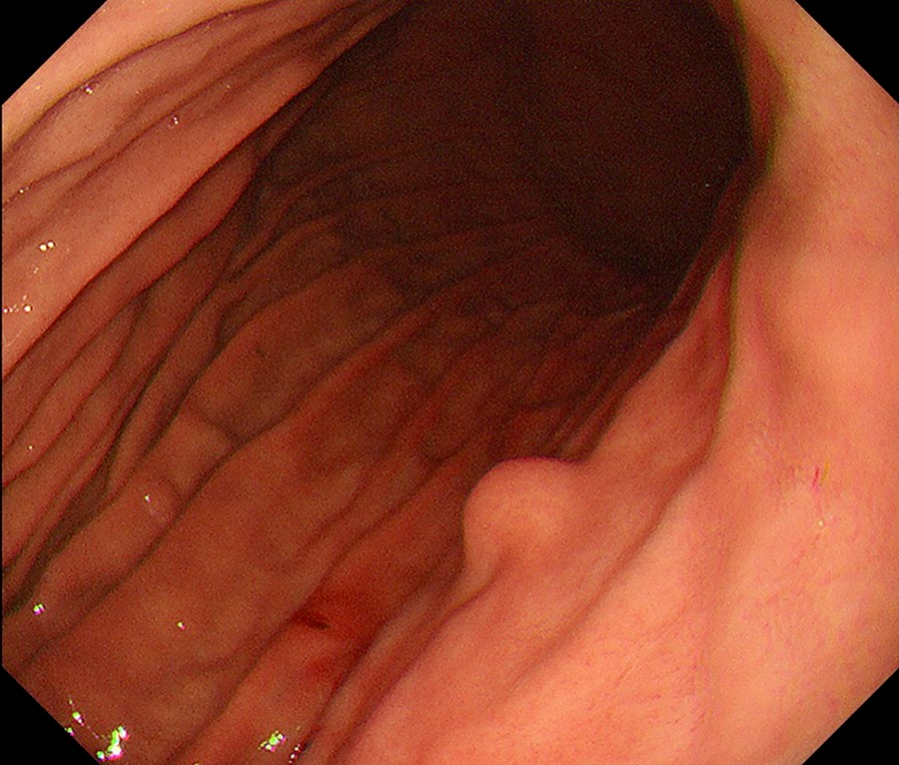
Upper gastrointestinal endoscopy revealed a 10 mm SEL in the posterior wall of the upper body of the stomach

A 49-year-old female patient with a history of diabetes mellitus (DM) presented with severe obesity. Her body weight was 92.7 kg, and her body mass index was 39.1 kg/m^2^. LSG was planned after completion of a 1-year course of conservative treatment for severe obesity. Preoperative, upper gastrointestinal endoscopy revealed a 10 mm SEL in the posterior wall of the upper body of the stomach (Fig. 1). Although neither a biopsy nor an endoscopic ultrasonography assessment was performed, a lymphangioma or lipoma was suspected on the basis of the endoscopic findings. If the SEL were left in the remnant stomach, follow-up endoscopy would be essential; if the SEL were to grow, it would lead to obstruction following LSG and would be difficult to resect after LSG. The patient wished to avoid frequent endoscopic follow-up examinations for the SEL and elected tumor resection during the LSG. Thus, in accordance with the patient’s wishes, SEL excision and LSG were performed simultaneously.

**Fig. 2:**
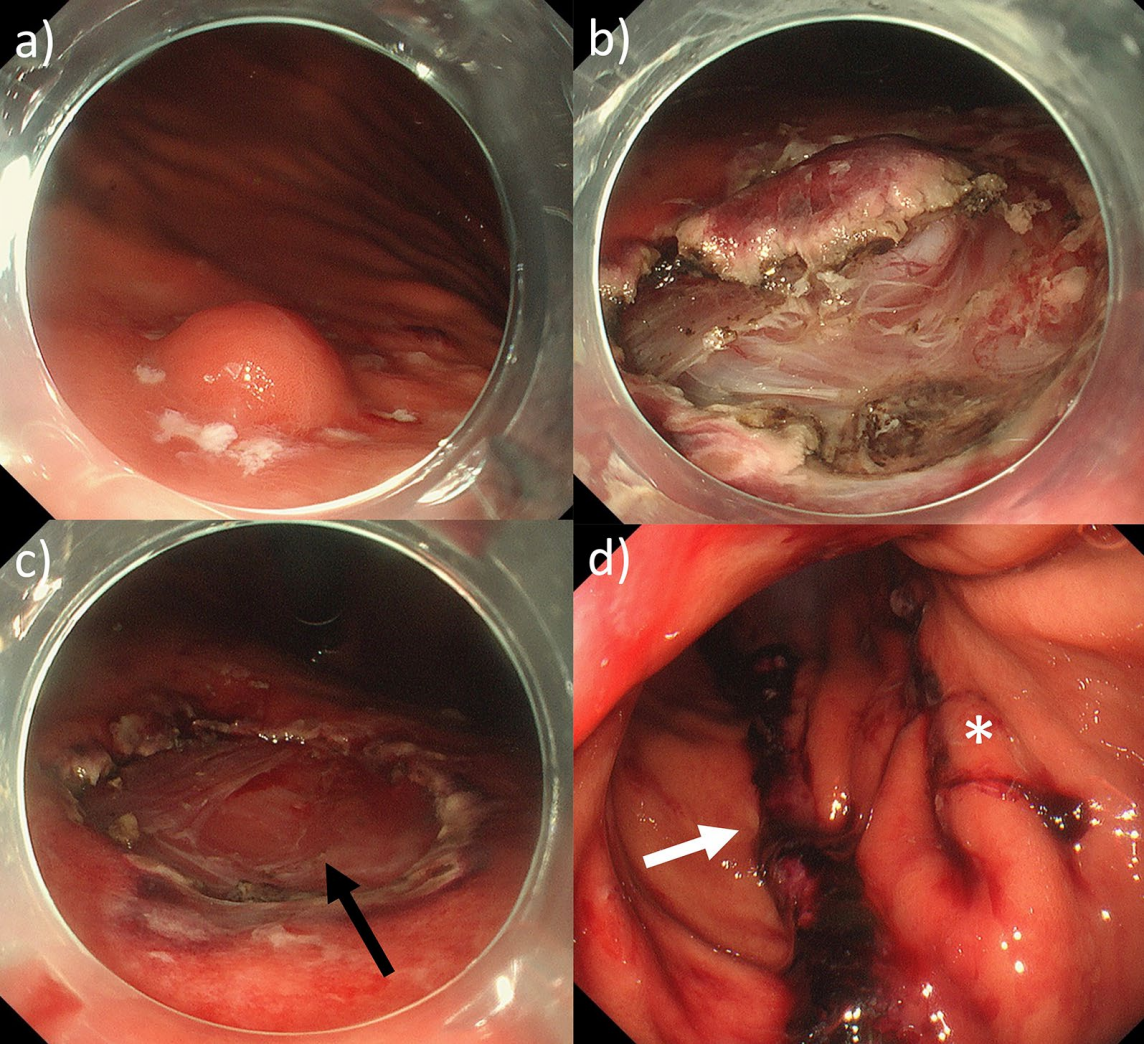
Intraoperative endoscopic findings. **a** Several dots were made around the tumor. **b** A circumferential incision was made to the depth of the submucosa around the tumor, and the MP layer at the bottom of the SEL was resected. **c** After endoscopic resection, the serosa was exposed (black arrow). **d** Following the LSG and LECS, care was taken to ensure that the staple line of the LSG (white arrow) did not overlap with the suture line of the LECS (*)

**Fig. 3:**
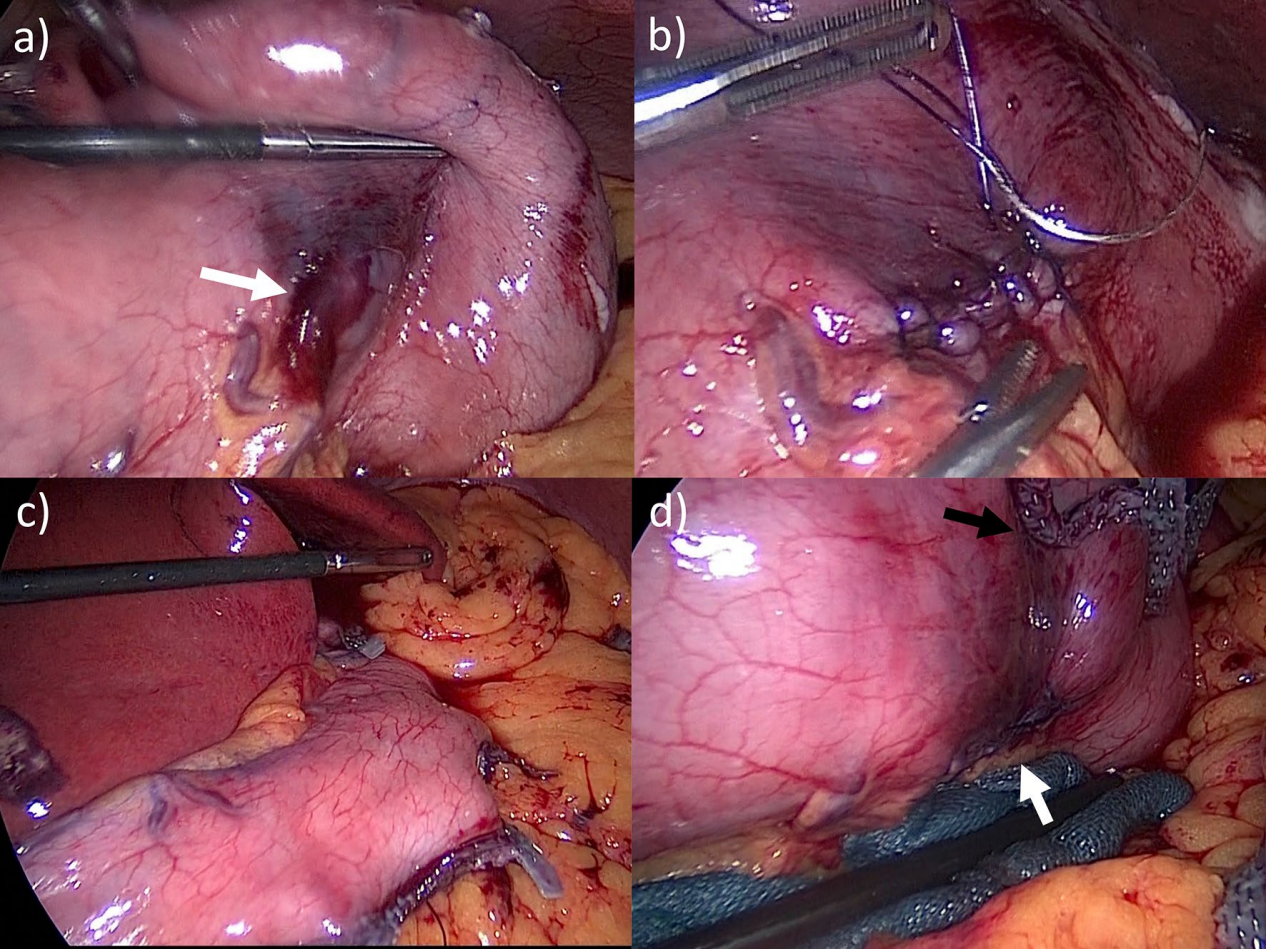
Intraoperative laparoscopic findings. **a** Excision of the SEL resulted in thinning of the gastric wall (arrow). **b** The thinned gastric wall was closed by hand-sewing. **c** Remnant stomach after LSG. **d** The staple line of the LSG (black arrow) did not overlap with the suture line of the LECS (white arrow)

After complete mobilization of the fundus, the posterior wall of the stomach was inspected. The SEL was not visible laparoscopically from the exterior of the stomach. Endoscopy revealed the location of the lesion in the remnant side of the stomach. Endoscopic resection via LECS was planned before the LSG. As the lesion was located deeply within the muscularis propria (MP) and near the serosa, the MP layer at its bottom was resected completely without damaging the capsule (Fig. 2a, b, c). After excision, the thinned gastric wall was closed by hand-sewing (Fig. 3a, b). Thereafter, the LSG was performed in the usual manner (Fig. 3c). The care was taken to ensure that the staple line of the LSG did not overlap with the suture line of the LECS (Figs. 2d, 3d). The total operative time was 312 min, and there was little blood loss. Based on a pathological analysis, which revealed a 9 × 4 × 7 mm nodule consisting of spindle cells, leiomyoma was diagnosed.

The postoperative course was uneventful, and the patient was discharged on postoperative day 6. The patient’s total weight loss was 23% at postoperative month 6, her DM remitted, and her HbA1c was 5.5% without treatment.

## Discussion

Severe obesity has rapidly increased over the past decade, and the use of bariatric surgery as its treatment has been on the rise. LSG is the most commonly performed bariatric procedure because it is relatively easy to perform, is effective, and has a low complication rate [2]. The increasing use of bariatric surgery, including LSG, has also increased the availability of bariatric surgery specimens for analysis. Finnell et al. reported a frequency of approximately 2% for diseases detected incidentally during bariatric surgery, such as benign ectopic pancreatic tissue, arteriovenous malformations of the small bowel, leiomyomas and gastrointestinal stromal tumors (GIST) of the stomach [1]. Furthermore, Crouthamel et al. reported an incidence of 1.2% for different forms of SEL excised after LSG; GIST were the most common (0.8%), followed by leiomyomas (0.3%) and schwannomas (0.1%) [3]. The incidence of GIST is thought to be higher in obese patients undergoing bariatric surgery (0.6–0.8%) than in the general population (0.0006–0.0015%) [4]. Moreover, the incidence of cancer is higher in obese patients [5].

Preoperative endoscopy assessment is important because it can detect pathologies, including SEL, and other clinically important findings before bariatric surgery, which will permit surgeons to choose the optimal surgical approach. The clinical practice guidelines of the European Association for Endoscopic Surgery on bariatric surgery provide a conditional recommendation for routine preoperative endoscopy, recognizing that selective endoscopy in patients with upper abdominal symptoms might be more appropriate [6]. As the incidence of gastric cancer is higher in Asians than in Caucasians, preoperative endoscopy should always be performed in Japan.

Simultaneous LSG and LECS could increase the risk of complications, including anastomotic leakage, so we considered performing the endoscopic resection of the SEL before the LSG. Donatelli G et al. reported a case series and good result of endoscopic resection of submucosal tumors prior to bariatric surgery [7]. However, the decision was finally made to perform these procedures simultaneously for two reasons: first, the scar resulting from the endoscopic resection of the SEL was predicted to be too near to, or overlap with, the LSG staple line, thereby increasing the risk of anastomotic leakage. On the other hand, performing the procedures simultaneously would allow the direction and position of the staple line to be adjusted according to the SEL site intraoperatively during the LSG. Second, if the endoscopic resection of the SEL were performed first, sufficient time would be needed for the inflammation and scarring resulting from the procedure to subside before performing the LSG. However, the patient had DM, and previous studies have found that LSG is more likely to improve or resolve this disease the shorter the disease duration [8]. Performing the endoscopic resection of the SEL before the LSG would lengthen the duration of the disease for the previously stated reasons. Therefore, the decision was made to perform the procedures simultaneously to minimize the duration of the disease state.

The intraoperative findings demonstrated that the SEL was located on the remnant side of stomach; thus, excision by LECS was performed simultaneously with the LSG. Although previous studies have detailed the excision of SEL and leiomyomas during LSG, there are thus far no reports of LECS for SEL being performed during LSG [9, 10]. The LECS combines endoscopic mucosal incision with laparoscopic surgery. Hiki et al. first reported the utility of the classical LECS using adequate cut lines for the safe excision of gastric SEL, such as GIST [11]. The major advantage of the LECS is that it requires less of the gastric wall to be resected than the conventional laparoscopic wedge resection using a linear stapler. Abe et al. described laparoscopically assisted endoscopic full-thickness resection (LAEFR), a modification of the classical LECS [12], in contrast to which the LAEFR uses endoscopy to create a circumferential mucosal or submucosal incision around the tumor while the deeper muscular and serosal layers are dissected endoscopically under laparoscopic guidance, and the gastric defect is closed by hand-sewing instead of being repaired with a linear stapler. In the present case, endoscopic full-thickness resection was unnecessary, but the MP layer at the bottom of the SEL was resected endoscopically, and the thinned gastric wall was closed by hand-sewing. LAEFR minimizes whole-layer excision while ensuring an adequate margin without the use of a linear stapler. These features prevent gastric stenosis and deformities of the remnant stomach after LSG. LAFER is thus an ideal method whenever LECS and LSG are to be performed simultaneously.

Care is required to prevent overlapping of the suture line of the LECS and the staple line of the LSG to prevent anastomotic leakage. However, while this may be achieved by adjusting the direction and position of the staple line during the LSG, this adjustment may result in a large divergence from the planned staple line, which can have two consequences: (1) reducing the size of the remnant stomach to the point of causing stenosis by deforming the remnant stomach; or (2) leaving too much of the remnant stomach as to nullify the weight reduction effect of the LSG. In cases where the staple line is predicted to diverge excessively from the planned position, surgeons should consider to exchanging the LSG for another procedure, for example, LSG to Roux-en-Y gastric bypass (RYGB) with resection of gastric remnant pouch [13].

Postoperative endoscopic follow-up is recommended for the early detection of malignancies or pathologies after bariatric surgery [14]. If any pathologies are detected, the surgical method best suited to managing the site and size of the pathology or previous bariatric surgery needs to be considered. Seki et al. reported a case of endoscopic submucosal resection of early gastric cancer after LSG with a duodenojejunal bypass [15]. Some authors have also reported cases of GIST in remnant stomach after RYGB for which a gastrectomy of the remnant was performed [16, 17].

## Conclusions

Herein, we reported performing LECS to excise a gastric SEL during a LSG for severe obesity. Surgeons should be prepared to manage incidentally detected pathologies before, during, and after bariatric surgery and to choose the surgical method most suitable to the individual patient.

## Data Availability

The data in this study are available from the corresponding author upon reasonable request.

## Abbreviations

SEL: Subepithelial lesion
LECS: Laparoscopic endoscopic cooperative surgery
LSG: Laparoscopic sleeve gastrectomy
DM: Diabetes mellitus
MP: Muscularis propria
GIST: Gastrointestinal stromal tumors
LAFER: Laparoscopically assisted endoscopic full-thickness resection
RYGB: Roux-en-Y gastric bypass

## Availability of data and materials

All the data generated or analyzed during this study are included in this article.
